# Cure of Epilepsy

**Published:** 1878-04

**Authors:** 


CURE OF EPILEPSY.—In the opinion of Kunze we possess in curare a remedy by means of which we may cure cases of epilepsy of long standing. He employs a solution of 7 grains of curare in 75 minims of water to which he adds two drops of hydrochloric acid. At intervals of about a week he injects beneath the skin 8 drops of this solution, and in various cases in which convulsions had occurred for several years he obtained a complete cure after 8 or 10 injections—*Lo Sperimentale.—Maryland Med. Jour.*

